# How does apathy impact exploration-exploitation decision-making in older patients with neurocognitive disorders?

**DOI:** 10.1038/s41514-023-00121-5

**Published:** 2023-10-30

**Authors:** Lyne Daumas, Raphaël Zory, Isabel Junquera-Badilla, Marion Ferrandez, Eric Ettore, Philippe Robert, Guillaume Sacco, Valeria Manera, Stephen Ramanoël

**Affiliations:** 1 Université Côte d’Azur, LAMHESS, Nice, France; 2 Université Côte d’Azur, CoBTeK, Nice, France; 3 Institut Universitaire de France, Paris, France; 4 Université Côte d’Azur, NeuroMod Institute, Nice, France; 5 Université Côte d’Azur, Centre Hospitalier Universitaire de Nice, service Clinique Gériatrique de Soins Ambulatoires, Centre Mémoire de Ressources et de Recherche, Nice, France; 6 Association Innovation Alzheimer, Nice, France; 7 Univ Angers, Université de Nantes, LPPL, SFR CONFLUENCES, 49000 Angers, France; 8 Sorbonne Université, INSERM, CNRS, Institut de la Vision, 17 rue Moreau, 75012 Paris, France

**Keywords:** Dementia, Signs and symptoms

## Abstract

Apathy is a pervasive clinical syndrome in neurocognitive disorders, characterized by a quantitative reduction in goal-directed behaviors. The brain structures involved in the physiopathology of apathy have also been connected to the brain structures involved in probabilistic reward learning in the exploration-exploitation dilemma. This dilemma in question involves the challenge of selecting between a familiar option with a more predictable outcome, and another option whose outcome is uncertain and may yield potentially greater rewards compared to the known option. The aim of this study was to combine experimental procedures and computational modeling to examine whether, in older adults with mild neurocognitive disorders, apathy affects performance in the exploration-exploitation dilemma. Through using a four-armed bandit reinforcement-learning task, we showed that apathetic older adults explored more and performed worse than non-apathetic subjects. Moreover, the mental flexibility assessed by the Trail-making test-B was negatively associated with the percentage of exploration. These results suggest that apathy is characterized by an increased explorative behavior and inefficient decision-making, possibly due to weak mental flexibility to switch toward the exploitation of the more rewarding options. Apathetic participants also took longer to make a choice and failed more often to respond in the allotted time, which could reflect the difficulties in action initiation and selection. In conclusion, the present results suggest that apathy in participants with neurocognitive disorders is associated with specific disturbances in the exploration-exploitation trade-off and sheds light on the disturbances in reward processing in patients with apathy.

## Introduction

Neurocognitive disorders are characterized by a decline in one or more cognitive domains that go beyond what is expected from normal aging. According to the DSM-5^1^, Mild neurocognitive disorders are characterized by a level of cognitive decline that requires compensatory strategies and accommodations to help maintain independence and perform activities of daily living, whereas Major neurocognitive disorders are characterized by cognitive impairment which affects autonomy in activities of daily living. In both Mild and Major neurocognitive disorders, neuropsychiatric symptoms are highly prevalent^2^, and among them, apathy is a multidimensional syndrome^3^ particularly pervasive, associated with numerous adverse outcomes^4,5^. Apathy is defined as a quantitative reduction in goal-directed behaviors and can manifest across several domains, including cognition, behavior, and emotion^6^. Clinically, apathy is manifested by difficulties in starting or maintaining the usual or desired activities, and it may be therefore complicated to identify it as some other symptoms can manifest similarly, such as fatigue^7^. From an etiological point of view, apathy may arise from dysfunction occurring at any step of the process of achieving goal-directed behaviors, including at the level of initiation, execution, and control of voluntary actions^8^.

Goal-directed behavior (i.e., voluntary actions toward outcome) relies on several interrelated processes, starting with an internal valuation step mainly supported by the ventral striatum (VS), the orbitofrontal cortex (OFC), the ventromedial prefrontal cortex (vmPFC) and the anterior cingulate cortex (ACC). The ACC attributes to stimuli of the environment, subjective values based on their hedonic or aversive potential, as well as the potential cost of action^9–11^. Goal-directed behavior relies on the capacity to represent and anticipate the action outcomes^12^, based on the previously learned associations between the actions and their consequences, which is mediated by the dopaminergic system^13^. The outcome value and cost of action are in this way accounted in the action selection phase, and the subsequent generation of behavior toward or away from stimuli. The decision-making processes of whether to engage or not in behavior are thus underpinned by weighting the prospective value of rewards (i.e., objects or events associated with positive outcomes that individuals pursue) against the amount of effort required to reach them^14^.

In apathetic patients, behavioral and neuroimaging studies suggest that these mechanisms of reward-based decision-making are disrupted, explaining at least in part their reduced willingness to engage in behavior^15–17^. More specifically, several lines of evidence have related apathy to lesions or dysfunctions in the brain circuit linking the frontal territories to the basal ganglia, notably in the vmPFC, the OFC, the ACC, and the VS^18–20^. Additionally, apathy has been related to dysfunction in the dopaminergic system^21^; and in line with this idea, some studies found improvement of apathy and reward sensitivity through dopaminergic medication^22^. For instance, Adam et al., described in their study, in apathetic patients exhibiting focal lesions in the basal ganglia connecting to the OFC and vmPFC, a reward insensitivity; and then its improvement following dopamine receptor agonist take^23^. Regarding experimental studies using choice tasks manipulating the level of effort and reward, it has been shown that apathetic individuals exhibited greater effort discounting (i.e., devaluation of subjective reward value by the amount of effort), so apathetic individuals needed larger rewards to engage in action^17,24^.

Regarding the exploration-exploitation decision-making, this dilemma is daily met by people and consists of choosing between a familiar option with a more predictable outcome and a less certain but potentially more rewarding option^25–27^. In other words, in an uncertain and changing environment where the values of potential options are unknown and can change over time, people must decide whether to utilize (“exploit”) the knowledge that has been accumulated or to explore new options. The neural evidence showed that exploration-exploitation is supported by the vmPFC and the rostral PFC, as well as the OFC, the ACC, and the VS^28–31^, with more particularly the involvement of the vmPFC in exploitation, and the rostral PFC in exploration^32^. Behaviorally, excessive exploitation restricts the possibility of obtaining new information, whereas too much exploration is associated with an inefficient use of acquired knowledge^33^. An optimal balance between exploration and exploitation is therefore required to maximize benefits, and it is also necessary to correctly learn and utilize the gained information. To this end, there is strong evidence that dopaminergic neurons play a central role in this type of learning by encoding the reward predictor errors (i.e., the differences between the experienced and expected outcome), which acts as a learning signal to update value predictions and then guide the action selection and future behaviors^34–36^.

In older adults, studies showed different exploration-exploitation patterns compared with younger adults^37,38^, and it has been suggested that it could be due to age-related modifications in the dopaminergic system, as well as to the decline in cognitive functions, notably in executive functions^39,40^. Indeed, executive functions are involved in decision-making, Their dysfunction is, by the way, also closely linked to apathy, and refers more particularly to impairment in the capacity to elaborate a plan of action (cognitive apathy)^8,41^. In Parkinson’s disease and schizophrenia, two pathologies with a high prevalence of apathy, some studies reported changes in solving the exploration-exploitation dilemma^42,43^. However, while it has been suggested that emotional apathy in dementia is underpinned by altered reward learning^44^, very few studies investigated direct links between apathy and exploration-exploitation decision-making^45^, notably in older adults with neurocognitive disorders. Yet, several pieces of evidence (e.g., common brain areas such as vmPFC, ACC, VS; dopaminergic system involvement, executive functions) raise the question of whether apathy in neurocognitive disorders affects exploration-exploitation decision-making. This investigation could be particularly relevant because this type of decision-making is performed daily by individuals and relies on socio-emotional and executive processing that is analogous to that of real-life goal-directed behavior (e.g., affective value attribution to environmental stimuli, integration of feedback, behavioral adjustments). This type of task could therefore be relevant to capture the complex nature of the multidimensional syndrome of apathy which includes emotional, cognitive, and behavioral components. Furthermore, in some cases, the standard tests of executive functions fail and/or are dissociated from real-life dysexecutive problems, which can be explained as the socio-emotional aspects are not considered. For instance, it has been observed that patients with OFC damage can be not cognitively impaired but exhibit severe daily life decision-making problems, with often behavior disorders. This functional decision-making task could provide new insight into apathy-related deficits which can have a direct impact on daily life, as well as regarding the underlying mechanisms of apathy, notably in neurocognitive disorders where apathy is highly prevalent and associated with faster clinical decline. Thus, the aim of this study was to examine the impact of apathy on exploration-exploitation decision-making in the population of older adults exhibiting a mild neurocognitive disorder, by comparing apathetic older adults with mild neurocognitive disorders vs. non-apathetic older adults with mild neurocognitive disorders.

## Results

Forty-six subjects from 60 to 89 years old were included (mean = 75.91 ± 6.54). According to the apathy diagnostic criteria, 19 were apathetic (age = 78.05 ± 5.05) and 27 were non-apathetic (age = 74.41 ± 7.12). Clinical and demographic data are presented in Table 1.

**Table 1:** Sample characteristics.

	All sample (*n* = 46)	Apathetic participants (*n* = 19)	Non-apathetic participants (*n* = 27)
Age (years)	75.91 ± 6.54	78.05 ± 5.05	74.41 ± 7.12
Sex (male)	17	9	8
Education level	1/8/10/27	1/4/4/10	0/4/6/17
Apathy Inventory	3.80 ± 4.52	8.74 ± 2.56	0.33 ± 0.73*
MFI	56.43 ± 19.42	71.05 ± 13.72	46.15 ± 16.03*
MoCA	24.78 ± 3.36	23.72 ± 3.55	25.48 ± 3.09
Stroop test	−0.27 ± 1.29	−0.40 ± 1.47	−0.18 ± 1.18
Corsi block-tapping test	14.02 ± 4.04	12.83 ± 3.62	14.88 ± 4.19
TMT-B	60.11 ± 31.97	46.67 ± 32.40	69.07 ± 38.89*

Education levels correspond, respectively, to primary, secondary middle school, secondary high school, and higher education.

*MFI* Multidimensional Fatigue Inventory, *MoCA* Montreal cognitive assessment, *TMT-B* Trail-making test-part B.

*p < 0.05; *p*-value from independent sample *t*-tests or Chi-squared tests.

### Exploration and performance on the bandit task

**Fig. 1:**
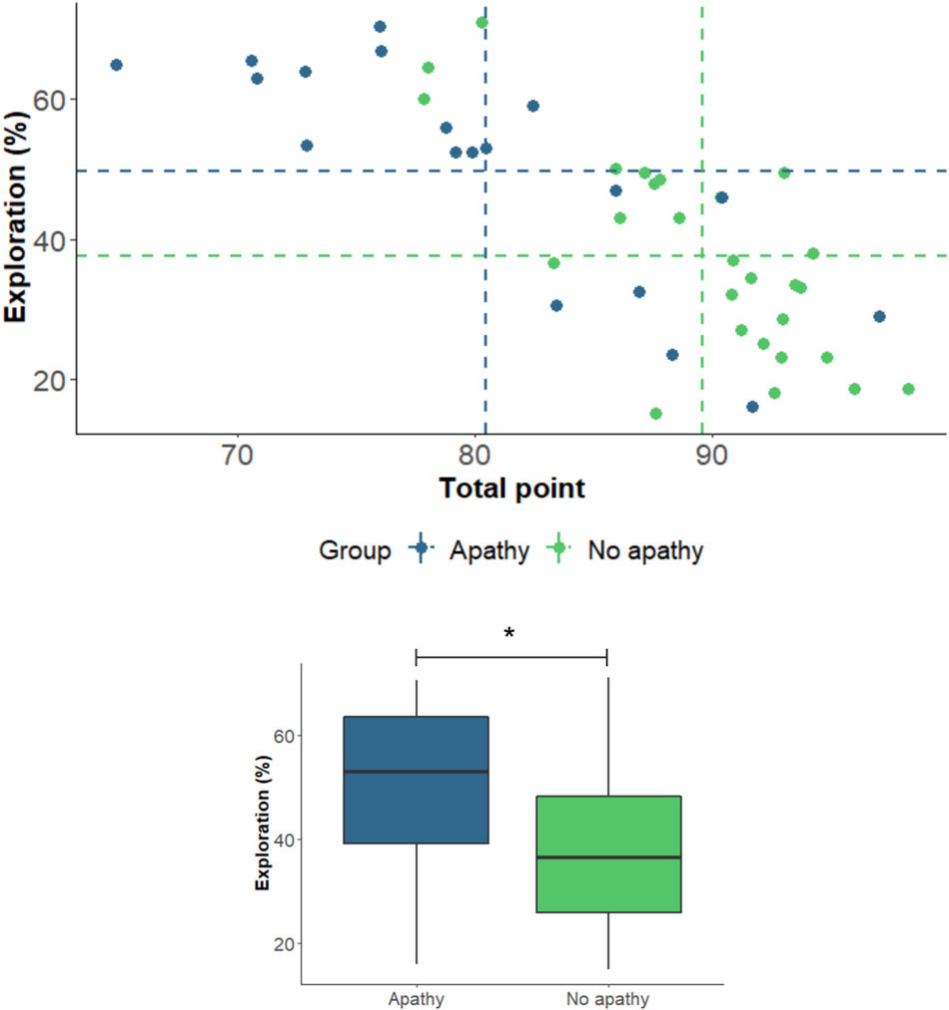
Percentage of exploration and total points earned on the bandit task in apathetic vs. non-apathetic participants. The horizontal dashed lines represent the mean of percentage exploration for the apathetic (blue) and non-apathetic participants (green). The vertical dashed lines represent the means of the total number of points earned, as measured by the percentage of theoretical maximum gain. The bottom bounds, the central lines, and the top bounds of the boxplots correspond, respectively, to the first quartiles (Q1), the medians, and the third quartiles (Q3). The minimums and maximums are represented by the ends of the whiskers. *p < 0.05 (MANCOVA and *t*-test).

**Fig. 2:**
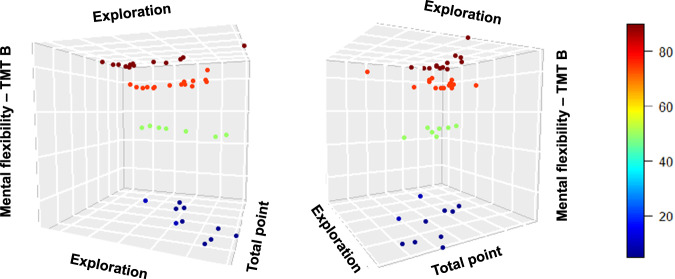
Percentage of exploration and total points earned on the bandit task according to the TMT-B score. Higher values of TMT-B (warmer colors) indicate better performance.

The percentage of explorative choices was higher in apathetic (49.79 ± 16.16, 95%CI [42.00, 57.58]) compared to non-apathetic participants (37.56 ± 14.7, 95%CI [31.74, 43.37]), however, their performance, as measured by the total points earned on the bandit task, was lower (80.44 ± 8.29, 95%CI [76.44, 84.44] vs. 89.60 ± 5.18, 95%CI [87.56, 91.65], Fig. 1). The MANCOVA revealed that these differences between groups were statistically significant after controlling for age, MFI, and TMT-B scores (*F*(2,39) = 5.62, *p* = 0.007, *η*^2^ = 0.22). No effect of gender was found (*F*(2,39) = 0.75, *p* = 0.47, *η*^2^ = 0.04) but the analysis revealed an effect of the TMT-B (*F*(2,39) = 4.37, *p* = 0.019, *η*^2^ = 0.18) such that worse performance was associated with more exploration (Fig. 2).

**Fig. 3:**
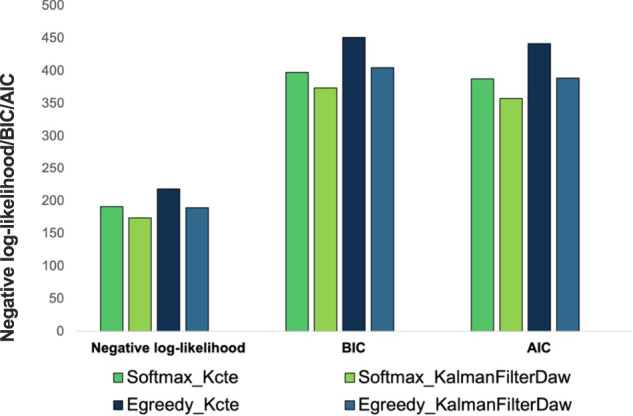
Comparison of models’ fits. Lower value of Negative log-likelihood, BIC, and AIC indicates better fitting.

Behavioral data were fitted with the four reinforcement-learning models. The model comparison revealed that the winning model was those with the “Kalman filter” learning rule and the “SoftMax” choice rule (Fig. 3), which considers that the bandit with the largest expected payoff had a higher probability of being chosen and the decision to explore others option had different probabilities, based on their previous payoff. Thus, the choice parameter *ß* adjusted for each participant, and giving information about the way they behaved on the task was used to examine whether apathetic patients used a different strategy. Consistent with the results above, the *ß* was lower among apathetic participants, indicating more explorative choices and the MANCOVA confirmed a main effect of apathy on the combined *ß* (apathetic group 0.118 ± 0.087, 95%CI [0.076, 0.160] vs. non-apathetic group 0.129 ± 0.087, 95%CI [0.101, 0.158]) and total point (apathetic group 80.44 ± 8.29, 95%CI [76.44, 84.44] vs. non-apathetic group 89.60 ± 5.18, 95%CI [87.56, 91.65]) variables after controlling for age, MFI and TMT-B scores (*F*(2,39) = 5.23, *p* = 0.009, *η*^2^ = 0.21), as well as the effect of the TMT-B (*F*(2,39) = 5.88, *p* = 0.006, *η*^2^ = 0.23). In addition, according to the *t*-tests, apathetic participants had significantly more missing responses compared to the non-apathetic participants (*t*(44) = 4.78, *p* < 0.001, mean difference = 9.01, 95%CI [5.26, 12.91], *d* = 1.43, 95%CI [0.76, 2.08]), and significantly higher latency of response (*t*(44) = 3.52, *p* = 0.001, mean difference = 185.56, 95%CI [79.24, 291.88], *d* = 1.05, 95%CI [0.42, 1.68]).

### Exploration-exploitation decision-making on the bandit task and cognitive performances

Among all participants, according to linear regressions, the TMT-B performance was significantly associated with the percentage of exploration after adjusting for age (*B* = 0.25, 95%CI [–0.40, –0.10], *ß* = –0.50, *p* = 0.002) such that better performance led to lower exploration. On the other hand, the MoCA and the Corsi scores were negatively associated with the percentage exploration (MoCA, *B* = –1.63, 95%CI [–3.04,–0.23], *ß* = –0.34, *p* = 0.023; Corsi, *B* = –1.48, 95%CI [-2.65,-0.32], *ß* = –0.37, – = 0.013) but no longer after adjusting for age (MoCA, *B* = –1.47, 95%CI [–3.02, 0.07], *ß* = –0.31, *p* = 0.06; Corsi, *B* = –1.40, 95%CI [–2.85,0.04], *ß* = –0.35, *p* = 0.056). The Stroop performance was not associated with the percentage of exploration (*B* = –1.49, 95%CI [-5.50, 2.52], *ß* = –0.121, *p* = 0.46).

## Discussion

Whether goal-directed behavior is worth pursuing or not is contingent upon the balance between effort and reward. Also, decision-making can be required to choose between exploiting a familiar option and exploring a lesser-known but potentially more rewarding option. The weighting effort-reward process has been proposed to be disrupted by apathy. However, very little is known regarding explorative-exploitative decision-making despite some evidence suggesting that it could also potentially be disrupted. The aim of the present study was to examine the explore-exploit decision-making in the apathy syndrome among older adults with mild neurocognitive disorders. As hypothesized, apathetic patients behaved differently than non-apathetic patients. They took a longer time to respond and explored more compared to the non-apathetic during the reinforcement-learning task. This pattern was associated with worse performance, as evidenced by lesser total point gain, thereby suggesting an excessive explorative and inefficient decision-making in apathetic individuals^33^.

The percentage of exploration was not significantly associated with the inhibition performance, nor with the memory performance after controlling for age, using the Stroop and Corsi tests. However, the TMT-B performance, assessing mental flexibility, was associated with explorative decision-making, such that lower performance was associated with higher exploration. This result suggests that reduced flexibility could be one explanation for high explorative choices found in apathetic patients with mild neurocognitive disorders compared with non-apathetic patients. Indeed, at the start, exploration is needed in order to learn about the values of available options, but then to be optimal, with the experience, using gained information, the more rewarding option must be exploited, thereby reducing the exploration. In an environment where options can change over time, to be efficient, people need to be able to switch their responses according to changes in the environment and, therefore, to adapt. Thus, a lack of flexibility to switch from the default explorative mode toward the exploitative mode can be one of the hypotheses to explain the prolonged exploration and worse performance in apathetic patients. These findings and this hypothesis of reduced flexibility are consistent with the apathy-related executive dysfunction^41^, and converge with results of the recent study of Scholl et al.^45^, where apathy has been linked to increased persisting in exploration, what they termed “decision inertia”. Participants with apathetic traits kept searching for new offers without considering that the appropriate strategy would be to stop the search. To describe this phenomenon of persistence in a sequence of searches for longer than appropriate, they employed the well-imaged expression to be “stuck in a rut”. But besides that, some studies have also reported higher exploration in individuals suffering from attention deficit hyperactivity disorders^46,47^. This suggests that the excessive exploration found in apathetic participants could also result from reduced attention, leading to reduced focus on the exploitation of the most relevant option. In line with this idea, a study conducted by Chau et al., used a visual scanning task and an eye-tracker to assess the attentional bias in apathy, in Alzheimer disease and found that apathetic participants had reduced duration of fixation on social images^48^. Thus, future studies should consider both mental flexibility and attention in the solving of exploration-exploitation decision-making among apathetic individuals.

One other complementary hypothesis that could explain the higher exploration and suboptimal decision-making on the reinforcement-learning task among apathetic participants compared with non-apathetic is a deficit in reward processing, including in the use of reward cues (which guide the behavior) and learning steps^49,50^; notably, as apathy has been associated with dysfunction in dopaminergic system^21^ which play a key role in learning^13^. Indeed, for efficiently solving the exploration-exploitation dilemma, it is necessary to correctly use the feedback, and learn and update the payoffs in order to identify the most rewarding option, thereby avoiding spending too much time in inefficient exploration. This hypothesis is consistent as apathy has been related several times to impaired reward processing^16,51,52^. For instance, Wong et al.^44^ demonstrated empirically, using a reward learning task, in older patients with neurocognitive disorders that emotional apathy was associated with difficulties in using socio-emotional rewards to guide behavior and impaired learning. Furthermore, several studies using gambling tasks, such as the Iowa gambling task (IGT), showed less efficient reward-based decision-making in apathy (i.e., lesser gain), which is consistent with present findings. For instance, a negative association between the level of apathy on the action initiation dimension and performance on the IGT was found in a study by Bayard et al. in patients with Alzheimer’s disease and mild cognitive impairment^53^. Similarly, in the study by Njomboro et al., apathetic patients with brain damage performed worse on the IGT by choosing more often options that led to a lesser gain in the long term^54^. Taken together, this evidence supports the idea that apathetic individuals may exhibit difficulties in using outcomes to identify and then exploit the most rewarding options. In other words, apathetic individuals may lose themselves in a multitude of ineffective explorations. In addition, it can be assumed that abnormalities in other steps of reward processing have contributed to this pattern of decision-making, such as reward insensitivity^12,55^. It could be, therefore, interesting in future studies to add tools allowing. objectively to measure the sensitivity to reward in probabilistic reward learning tasks. Last, current findings could be explained by the relying of exploitation on the structures involved in anticipation, valuation, and experience of rewards comprising notably the mesocorticolimbic dopaminergic pathway and the vmPFC^32^, which are also found disrupted in apathy^15,18^.

In addition, to be more explorative, to perform the four-armed bandit task, apathetic participants took longer time to make their choice and more often failed to respond in the allotted time. These results may reflect the difficulties in action initiation^3^ and difficulties in making a choice^56^ which are both clinical features of apathy. Longer times to perform decisional tasks or initiate motor actions have been previously reported in apathetic individuals. For instance, using a grasping task involving manual motricity, Manera et al.^57^ recorded longer times to start the action, which has been suggested as an index of deficit in action initiation. In a study of Martinez-Horta et al., using a gambling task, reduced reaction times were also recorded^52^. Here, using the bandit reinforcement-learning task, these difficulties in making choices seem to have been expressed by both longer response times and inertial and suboptimal decisions. These results are, therefore, in line with clinical aspects regarding this feature that is explicitly formulated in clinical diagnostic criteria as follows “takes longer to make choices when different alternatives exist (e.g., selecting TV programs, preparing meals, choosing from a menu, etc.)”^56^.

To summarize, in the bandit task, as in more ecological situations, optimal decision-making involves the capacity to adapt one’s actions according to environmental changes. This needs mental flexibility involved to be able to update new information and to manage attention efficiently. According to the “ambidexterity hypothesis”^58^, the higher the ability to balance exploration-exploitation, the better the performance. Not enough exploration deprives people of information about their environment, whereas too much exploration may lead to inefficient decision-making^33^. In the present study, this exploration-exploitation decision-making has been examined among older apathetic patients. For that, we notably used reinforcement-learning models, as they are widely used to capture the computational process of based-feedback learning and to describe how individuals behave and make decisions^59,60^. Like in numerous studies, the SoftMax choice rule from the computational model had the best fit with the behavioral data of participants, and therefore the *ß* free parameter was used to identify the individual specificities regarding the exploration^60^. Results showed higher exploration choices, suboptimal decision-making, and longer time of response in apathetic individuals compared with non-apathetic. Taken together, results fit well with clinical characteristics such as retardation, limited interest, and difficulties in making choices and suggest that the exploration-exploitation dilemma represents an interesting framework in apathy research, especially in neurocognitive disorders where apathy is highly prevalent. Indeed, it allowed us to identify a specific behavioral phenotype associated with apathy in mild neurocognitive disorders, thereby providing a new approach for capturing a specific way to behave (behavioral level). It could also help to provide insight into potential disrupted mechanisms (explanatory process level)^61^. However, here, some factors that might influence findings, such as attention, have not been measured, and no objective measure to assess the underlying mechanisms has been used. Thus, it could be interesting to add eye tracking to further investigate the role of attention and reward sensitivity. In addition, further behavioral studies should specifically examine these potentially disrupted mechanisms, using, for instance, neuroimaging or pharmacologic interventions (e.g., examine the association between brain activation in orbital-ventromedial prefrontal cortex and exploration-exploitation decision-making in apathetic individuals, assess the effects of the dopaminergic drug in explorative behavior among apathetic individuals), and this in various population (e.g., young adults, healthy older adults, older adults with other neuropsychiatric diseases such as schizophrenia). Furthermore, considering the previous studies showing disruption of effort-reward-based decision-making in apathy and the present results suggesting an imbalance in exploration-exploitation trade-off, it could be relevant to examine whether other factors of decision-making are affected by apathy, such as the temporal proximity to obtain a reward that also influences the subjective value attributed to options and the subsequent action selection. This could be especially relevant as it has been suggested that delay, effort, and probability decision-making are closely related and underpinned by some common underlying brain area^62,63^. Present results provide new insight. These research works on apathy and decision-making could in perspective be used to develop tasks for clinical practice, for aiding apathy detection. Nevertheless, it must be noticed that one of the limitations of the study is the absence of a healthy aged matched control group, so future studies should include one in order to assess the role of neurocognitive disorders.

## Methods

### Participants

Forty-six patients aged 65 years and older with mild neurocognitive disorders (NCD) were recruited in a Research Memory Center. Indeed, in order to examine the effect of apathy on exploration-exploitation decision-making in the NCD, we included and compared older adults with mild NCD exhibiting apathy vs. older adults with NCD without apathy. Here, healthy older adults without NCD were not included (but this is the subject of another ongoing study that includes the general population, including older adults, where apathy is assessed using self-reported scales). The mild NCD were identified by physicians according to the Diagnosis and Statistical Manual of Mental Disorders, 5th edition criterion (DSM-5^1^) based on clinical interviews and using the neuropsychological testing performed by neuropsychologists. Participants had to be able to speak and understand French. Non-inclusion criteria were (1) the presence of current uncontrolled major psychiatric disorders (e.g., major depressive disorder), (2) visual, auditive, and motor impairments not allowing to perform the task, (3) incapacity to provide the consent to participate. In the sample, 19 patients were identified as apathetic using the Apathy Diagnostic Criteria^3^. The study was performed in accordance with the declaration of Helsinki and approved by the ethical committee “Comité de Protection des Personnes (CPP) Ile de France IV”, N° IDRCB: 2021-A03126-35. Written informed consent to participate in the study was obtained from all participants. An a-priori power analysis was performed using the G*Power software, in order to compare the exploration-exploitation decision-making in apathetic vs. non-apathetic participants. The sample size was estimated to be 42 subjects (subjects (*α* = 0.05, Power = 0.80, effect size *d* = 0.8).

### Measures

#### Apathy and fatigue assessment

Apathy diagnostic criteria^3^ was used by clinicians to identify apathetic patients. In addition, the clinician version of Apathy Inventory (AI)^64^ was administered. The AI assesses emotional blunting, loss of initiative, and loss of interest. Each domain is scored from 0 (“no problem”) to 4 (“major problem”). The total score ranges from 0 to 12, with a higher score indicating greater apathy.

Fatigue was assessed using the Multidimensional Fatigue Inventory (MFI)^65^, a 20-item self-report questionnaire. Items are scored on a five-point Likert scale, and total scores range from 20 to 100 such that a higher score indicates greater fatigue.

#### Cognitive assessment

The Montreal Cognitive Assessment^66^ (MoCA) was administered to assess global cognitive functioning. Scores ranged from 0 to 30 with the higher score indicating better performance. In addition, as executive functions play a crucial role in problem-solving and decision-making as well as in apathy, and they have been related to the resolving of such task^67,68^, the several aspects of executive functions that include the working memory (also called “updating”), the cognitive flexibility and the inhibitory control^69^ were assessed using specific tests. More specifically, the Corsi block-tapping test^70^ was used to assess visuo-spatial short-term memory and working memory (i.e., capacity to retain information while performing mental operations on that information). A higher score indicated a higher performance. Cognitive flexibility (i.e., capacity to switch a course of thought or action in order to adapt to changes of situation) was assessed using part B of Trail making test^71^ (TMT-B), and participants were classified in percentile using normative data. The Stroop test^72^ assessed the inhibitory control (i.e., capacity to inhibit dominant responses to stimuli in order to select one more appropriate), and the z-score was used.

#### Exploration-exploitation decision-making task

**Fig. 4:**
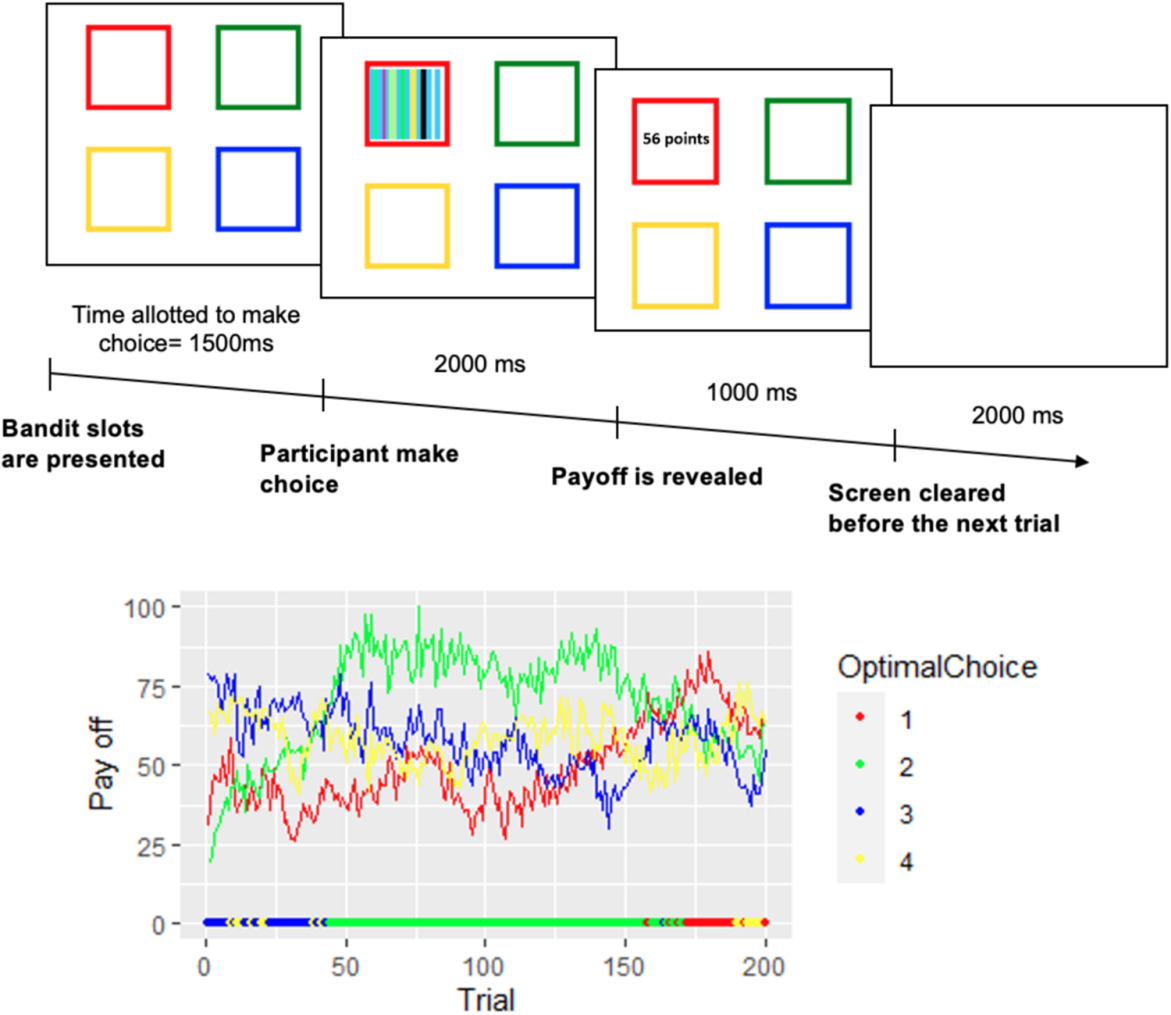
Four-armed bandit task. Experimental task used to assess the exploration–exploitation decision-making trade-off. Illustration of the timeline within a trial, and evolution of gain as a function of trial for each option (red, green, yellow, blue). At the bottom, for each trial, the best option (with the best pay off on the current trial) is represented (red, green, yellow or blue dot/option).

To assess the exploration-exploitation decision-making, a modified version of the “four-armed bandit task” from Daw et al.^59^ was performed on a touch-screen computer (Fig. 4) using the Inquisit software (Millisecond, United States). The task included 200 trials divided into two blocks of 100 trials and separated by a 60-s break. Each trial started with a presentation of four different colored squares representing the four choice options. The participants had a maximum of 1.5 s to choose one of the four squares by finger touching the screen. Then, the number of points earned was displayed in the chosen colored square. However, the payoff of the non-chosen was not displayed. The payoff for each trial ranged from 1 to 100 points, and the objective for the participant was to accumulate as many points as possible across trials. If no choice was made within the given time, a red cross was displayed in the center of the screen, meaning a missed trial with no point earned. Participants were informed during the instruction phase that the number of points paid off by the four squares gradually and independently changed from trial to trial.

The number of points paid off by the slot machine *i* on trial *t* was generated in a Gaussian distribution with a standard deviation of 4 and an approximate mean *μ*_i,t,_ which changed from one trial to the next as below:

1 $${\mu }_{i},{t+1}={{\lambda }}{\mu }_{{i}},{{t}}+(1-{\lambda }){{\theta }}+{{\nu }}$$

The decay parameter *λ* was 0.9836, and the decay center *θ* was 50. The diffusion noise *ν* was zero-mean Gaussian with a standard deviation of 2.8. The values of rewards were displayed only by selecting the slot; thus, by selecting different alternatives, the participant can learn about the options and use this information to modify their decisions in order to improve gains. Each choice was classified as explorative if participants selected a slot that did not have the highest known payoff at this point and as exploitative if the selected slot was the one with the maximum payoff known at this point. The percentage of explorative choices was computed as well as the total score, as measured by the total number of points earned to the theoretical maximum gain ratio. The number of missing responses and time to choose was also recorded.

### Reinforcement-learning model

Behavioral data was modeled using four reinforcement-learning models by combining two different learning rules (*k* constant vs. Kalman filter) with two different choice rules (SoftMax vs. ε-greedy). The learning rules computed and updated the expected payoffs (${\hat{\mu }}_{i,t}$) of each option (*i)* on each trial (*t)* based on previous choices and obtained rewards and then, based on these estimations, the choice rules computed the probability of each bandit to getting selected (${P}_{i,t}$). These approaches were used as the ε-greedy and k constant are considered as a basic rule in the reinforcement-learning model, and because the SoftMax is a major one it is largely used in studies, including in those involving older adults^60^.

With the first learning rule (*k* constant) tested, the estimated payoff of the chosen *c* bandit on trial *t* was updated by the obtained reward *r* as follows:

2 $${\hat{\mu }}_{c,t\,}^{{{\mathrm{post}}}}={\hat{\mu }}_{c,t\,}^{{{\mathrm{pre}}}}+{\hat{\kappa }\delta }_{t}$$

with ${\delta }_{t}$ the prediction error on trial *t* corresponding to ${r}_{t}-{\hat{\mu }}_{c,t}$ and $\hat{\kappa }$ the learning rate parameter. For the unchosen bandit, the estimated payoffs do not change.

For the second learning rule tested, the Kalman filter as implemented by Daw et al. (2006)^59^ was run. In this Bayesian model, in addition to tracking the mean payoff (${\hat{\mu }}_{i,t}$), the model computed the uncertainty (${\hat{\sigma }}_{i,t}^{2}$), which corresponded to the variance of the distribution of the expected reward, and determined the learning rate from trial by trial (${\hat{\kappa }}_{t}$). When the participant chose a bandit *c* on trial *t*, the posterior estimated distribution with mean (${\hat{\mu }}_{c,t}^{{{\mathrm{post}}}}$) and variance (${\hat{\sigma }}_{c,t}^{2{{\mathrm{post}}}}$) were updated as follows:

3 $${\hat{\mu }}_{c,t\,}^{{{\mathrm{post}}}}={\hat{\mu }}_{c,t\,}^{{{\mathrm{pre}}}}+{\hat{\kappa }\delta }_{t}$$

with ${\delta }_{t}$ the prediction error on trial *t* corresponding to ${r}_{t}-{\hat{\mu }}_{c,t}$

4 $${\hat{\sigma }}_{c,t}^{2{{\mathrm{post}}}}=\left(1-{\hat{\kappa }}_{t}\right){\hat{\sigma }}_{c,t}^{2{{\mathrm{pre}}}}$$

with ${\hat{\kappa }}_{t}$ the learning rate computed as ${\hat{\sigma }}_{c,t}^{2{{\mathrm{pre}}}}$/(${\hat{\sigma }}_{c,t}^{2{{\mathrm{pre}}}}+{\hat{\sigma }}_{o}^{2}$) where ${\hat{\sigma }}_{o}^{2}$ is the estimated observation variance.

For all bandits, the distribution of the expected payoffs (prior mean and variance) on the subsequent trial were updated as follows:

5 $${\hat{\mu }}_{i,t+1}^{{{\mathrm{pre}}}}=\hat{\lambda }{\hat{\mu }}_{i,t\,}^{{{\mathrm{post}}}}+(1-\hat{\lambda })\hat{\theta }$$

6 $${\hat{\sigma }}_{i,t+1}^{2{{\mathrm{pre}}}}={\hat{\lambda }}^{2}{\hat{\sigma }}_{i,t}^{2{{\mathrm{post}}}}+{\hat{\sigma }}_{d}^{2}$$

Then, using the SoftMax choice rule, the resulting probabilities (${P}_{i,t}$) of choosing option (*i*) on trial (*t*) were computed according to:

7 $${P}_{i,t}=\frac{\exp (\beta {\hat{\mu }}_{i,t\,}^{{\mathrm{pre}}})}{{\sum }_{j}\exp (\beta {\hat{\mu }}_{i,t\,}^{{\mathrm{pre}}})}$$

with *ß* the parameter giving information about the explorative behavior of participant such that lower *ß* indicate more exploration.

The second choice rule was the ε-greedy, as used by Daw et al. (2006). With this model, the probabilities (${P}_{i,t}$) of choosing option (*i*) on trial (*t*) were computed according to:

8 $${P}_{i,t}=\varepsilon +(1-4\varepsilon )\frac{\exp \left(\left(\frac{100}{\sqrt{\sum k{\mu }_{i,k}}}\right){\mu }_{i,t}\right)}{{\sum }_{j}\exp \left(\left(\frac{100}{\sqrt{\sum k{\mu }_{j,k}}}\right){\mu }_{j,t}\right)}$$

with *ε* giving information about the exploitative behavior of participant such that lower *ε* indicate more exploitation.

Thus, the ε-greedy rule considered that the greedy choice, the one with the largest anticipated pay-off, had a higher probability to be chosen, and the other, non-greedy, options were uniformly explored whereas, with the SoftMax rule, the decision to explore other options had different probability and were based on their previous payoff. While the percentage of exploration was based on the seen payoff (such that the choice of the bandit with the highest payoff seen at this time corresponded to exploitation), in the models, the classification was based on the estimated pay-off, as computed in the learning rule, (such that the choice of the bandit with the highest estimated pay-off correspond to exploitation). The free parameters of models (*ß* or *ε*) were computed to fit with the data of each participant in order to give information about the way each of them behaved.

In order to identify the best model, models’ fits were compared using the negative log-likelihood, the Akaike Information Criterion (AIC), and the Bayesian Information Criterion (BIC).

### Statistical analysis

To examine behavioral performance on the bandit task and the associated efficiency in apathetic vs. non-apathetic participants with mild neurocognitive disorders, multivariate analyses of covariance (MANCOVA) with apathy status as a predictor factor were conducted. One was run with the percentage of exploration and the total number of points as dependent variables. The other one was conducted with the total number of points and the estimated parameter from the best reinforcement model (*ß*, *ε*). All were adjusted by age, cognitive performance, and fatigue level. Effect sizes were computed using the partial eta squared (*η*^2^) where a value of 0.01, 0.06, and 0.14 corresponded, respectively, to a small, medium, and large effect size. For the number of missing responses and the chosen time, one-sided Student *t*-tests were performed, and Cohen’s *d* was used to calculate the effect size. To examine the associations between cognitive performance and exploration behavior, linear regressions were conducted. For all tests, the alpha level was set at 0.05.

### Reporting summary

Further information on research design is available in the Nature Research Reporting Summary linked to this article.

### Supplementary information

Reporting summary

## Data Availability

The datasets generated and analyzed during the current study are available from the corresponding authors upon reasonable request.
